# Evaluation of the position and morphology of tongue and hyoid bone in skeletal Class II malocclusion based on cone beam computed tomography

**DOI:** 10.1186/s12903-021-01839-y

**Published:** 2021-09-27

**Authors:** Wener Chen, HungEn Mou, Yufen Qian, Liwen Qian

**Affiliations:** Department of Orthodontics, Shanghai Ninth People’s Hospital, College of Stomatology, Shanghai Jiao Tong University School of Medical, Shanghai, 200011 China

**Keywords:** Tongue posture, Hyoid bone position, Morphology, Malocclusion, Cone beam computed tomography

## Abstract

**Background:**

The aim of the study was to analyze the morphology and position of the tongue and hyoid bone in skeletal Class II patients with different vertical growth patterns by cone beam computed tomography in comparison to skeletal Class I patients.

**Methods:**

Ninety subjects with malocclusion were divided into skeletal Class II and Class I groups by ANB angles. Based on different vertical growth patterns, subjects in each group were divided into 3 subgroups: high-angle group (MP-FH ≥ 32.0°), average-angle group (22.0° ≤ MP-FH < 32°) and low-angle group (MP-FH < 22°). The position and morphology of the tongue and hyoid bone were evaluated in the cone beam computed tomography images. The independent Student’s *t*‐test was used to compare the position and morphology of the tongue and hyoid bone between skeletal Class I and Class II groups. One-way analysis of variance (ANOVA) was used to compare the measurement indexes of different vertical facial patterns in each group.

**Results:**

Patients in skeletal Class II group had lower tongue posture, and the tongue body was smaller than that of those in the Class I group (*P* < 0.05). The position of the hyoid bone was lower in the skeletal Class II group than in Class I group (*P* < 0.05). The tongue length and H-Me in the skeletal Class I group with a low angle were significantly larger than those with an average angle and high angle (*P* < 0.05). There was no significant difference in the position or morphology of the tongue and hyoid bone in the skeletal Class II group with different vertical facial patterns (*P* > 0.05).

**Conclusion:**

Patients with skeletal Class II malocclusion have lower tongue posture, a smaller tongue body, and greater occurrence of posterior inferior hyoid bone position than skeletal Class I patients. The length of the mandibular body in skeletal Class I patients with a horizontal growth type is longer. The position and morphology of the tongue and hyoid bone were not greatly affected by vertical facial development in skeletal Class II patients.

## Background

The growth of the craniomaxillofacial system is influenced by both genetic and environmental factors. Many studies have shown that the neuromuscular equilibrium plays an important role during maxillary-mandibular growth, and may affect the establishment of occlusion relationship [1, 2].

Skeletal class II malocclusion is a common dentofacial deformity. In addition to the disorder of anteroposterior occlusal relationship, it is also accompanied by the disharmony of the size of the maxilla and mandible [3]. Although some studies have pointed out that Class II malocclusion featuring mandibular deficiency occurs in response to function, the degree of interplay is still a matter of discussion. To assess environmental effects on the development of class II skeletal malocclusion, the knowledge of its association with given environmental factors, i.e., tongue posture and the position of the hyoid bone, would be useful. Liégeois found that the shape, position and mobility of the tongue are related to the dental arch forms and occlusion [4]. The position of hyoid bone relative to the skull base and mandible can be used as an index of tongue posture and function [5]. Abnormalities in either function or position of the tongue and the hyoid bone can lead to changes in the surrounding alveolar structures [6]. Thus, it is important to consider the etiological factor at the beginning of orthodontic treatment so that the efficacy and long-term stability of treatment could be enhanced.

Cone beam computed tomography (CBCT) provides a reliable and accurate method to evaluate the craniomaxillofacial structure and the position and morphology of tongue and hyoid bone in patients [7]. However, CBCT scans have rarely been used to comprehensively investigate tongue and hyoid bone in patients with skeletal class II malocclusion. Therefore, the aim of the present study was to analyze the morphology and position of tongue and hyoid bone in skeletal Class II patients with different vertical growth patterns by CBCT to provide reference for clinical diagnosis and predict the possible changes in craniomaxillofacial growth direction.

## Methods

Ethical approval for this study was obtained from the Ethical Committee of the local institution, and written informed consent was obtained from each patient. All methods involved below were performed in accordance with the relevant guidelines and regulations.

### Sample collection

The subjects were divided into 2 groups according to their ANB angles: class II malocclusion patients (ANB > 4.5°) and class I malocclusion patients (0.7° ≤ ANB ≤ 4.5°), who composed the control group. The Class I and Class II groups consisted of 18 males and 27 females (average age, 15.4 ± 3.2 years), and 17 males and 28 females (average age, 16.6 ± 2.7 years), respectively. According to the Frankfort mandibular plane angle, the patients in each group were divided into 3 subgroups: high-angle type (MP-FH ≥ 32.0°), average-angle type (22.0° ≤ MP-FH < 32°) and low-angle type (MP-FH < 22°). The inclusion criteria were as follows: no previous treatment for tonsillectomy or adenoidectomy, no history of orthodontic or orthognathic surgical treatment, no history of temporomandibular joint disorders, no craniofacial or growth abnormalities, no missing permanent teeth, and acceptable oral hygiene without obvious periodontal disease.

### CBCT processing

For CBCT scanning, each patient was seated in an upright position and instructed to keep their teeth in maximum intercuspation, with the lips in light contact and tongue in a resting position. The Frankfort horizontal plane (FH plane) of the patients was kept parallel to the floor. Each patient was asked to not move his or her head or swallow during the scanning process. The CBCT device (VG0910 3S, NewTom, Italy) was set to a maximum of 110 kV, maximum of 15 mA, exposure time of 5.4 s and voxel dimension of 0.39 mm. All CBCTs were taken by the same operator. CBCT images were then saved as DICOM (digital imaging and communications in medicine) files. The DICOM files were imported into Mimics 20.0 software (Materialise, Leuven, Belgium), and the images in the axial, coronal, and sagittal planes were automatically generated. The mid-sagittal images were selected to locate the tongue posture using the nasion (N), anterior nasal spine (ANS), posterior nasal spine (PNS) and basion (Ba). The shape and position of the tongue and hyoid bone were then measured and compared.

### Labeling of landmarks and planes

Landmarks: sella (S), nasion (N), basion (Ba), tip of tongue (TT), deepest point of the epiglottis (EP), most antero-inferior point on the corpus of the third cervical vertebra (C3), menton (Me), most antero-superior point on the body of the hyoid bone (H), and intersection point of vertical line of C3-Me through H with line C3-Me (H’).

Reference planes: line between TT and EP (reference plane of tongue), line from S to N (SN plane), line with an angle of 15° to SN plane through S (PS plane), and line from S vertical to PS plane (VPS plane).

### Evaluation of tongue posture

**Fig. 1:**
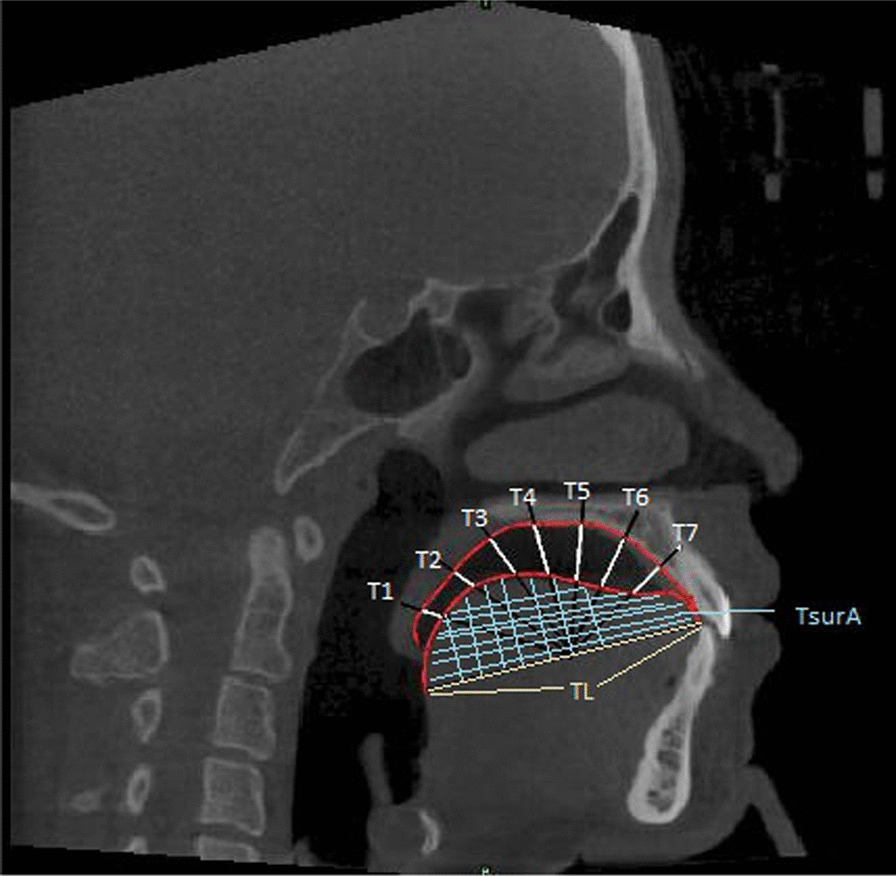
Assessment of tongue posture and morphology (T1–T7: distances between the tongue and plate; TL: tongue length; TsurA: sagittal sectional area of tongue)

Tongue posture was assessed using the method described by Graber et al. [8]. A template with an inscribed millimeter scale was used to assess the tongue position relative to the palate. Taking the midpoint of the line between TT and EP as center, the reference plane of the tongue was rotated clockwise by 30°, 50°, 70°, 90°, 110°, 130° and 150°. The contours of the dorsum of the tongue and the palate were traced, and seven distances (T1–T7) between the tongue and palate were recorded at 30°, 50°, 70°, 90°, 110°, 130° and 150° (Fig. 1).

### Evaluation of tongue morphology

Tongue length (TL): linear distance between TT and EP; Sagittal sectional area of tongue (TsurA): closed area between the reference plane of tongue and the dorsum of tongue (Fig. 1).

### Evaluation of the position of hyoid bone

**Fig. 2:**
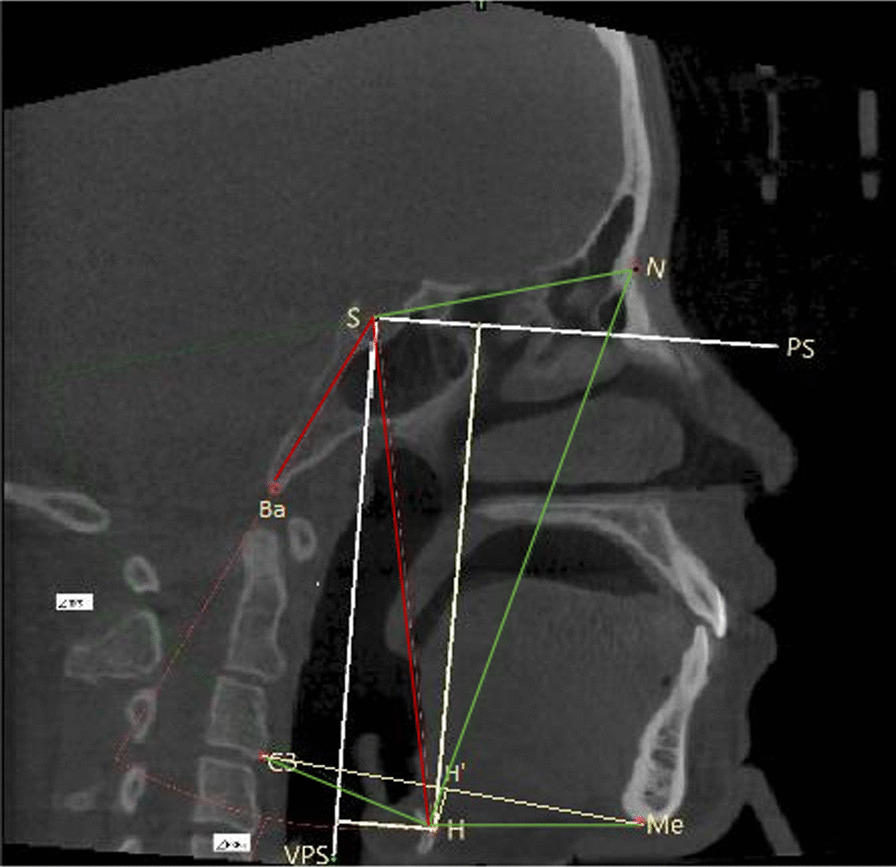
Assessment of the position of hyoid bone: (1) C3-Me; (2) C3-H; (3) H-Me; (4) H–H’; (5) H–X(the vertical distance from H to the VPS plane); (6) H–Y (the vertical distance from H to the PS plane); (7) H–N–S (angle formed by H–N line and N–S line); (8) H–S–Ba (angle formed by H–S line and S–Ba line)

The hyoid bone position was evaluated using six linear parameters C3–Me, C3–H, H–Me, H–H’, H–X, and H–Y, as well as two angular parameters, H–N–S and H–S–Ba (Fig. 2).

### Evaluation of hyoid bone morphology

**Fig. 3:**
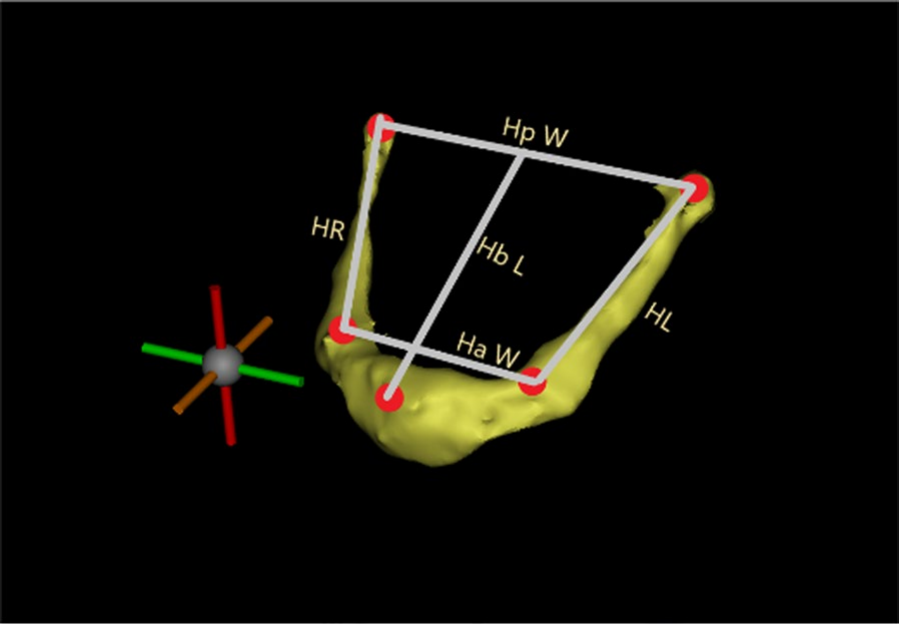
Assessment of hyoid bone morphology: (1) Ha W; (2) Hp W; (3) Hb L; (4) HR; (5) HL

After three-dimensional reconstruction of the hyoid bone model, the measurement parameters of hyoid bone morphology are as follows: the anterior width of the hyoid bone (Ha W): the horizontal distance between the bilateral intersections of the hyoid body and thyrohyal; the posterior width of the hyoid bone (Hp W): the horizontal distance between the bilateral ends of the thyrohyal; the hyoid body length (Hb L): the vertical distance from H to the line between bilateral ends of thyrohyal; the length of right thyrohyal (HR); and the length of left thyrohyal (HL) (Fig. 3).

### Statistical analysis

Statistical analyses were performed using SPSS 23.0 for Windows software package. The independent Student’s *t*‐test was used to detect significant differences in the position and morphology of the tongue and hyoid bone between the two groups. One-way analysis of variance (ANOVA) was used to compare the measurement indexes of different vertical facial patterns in each group. The significance level was set at *P* < 0.05.

## Results

### Tongue measurements in two groups

The descriptive statistics for the tongue measurements of the two groups are shown in Table 1. The comparison of mean tongue-to-palate distances between the two groups showed statistically significant differences (*P* < 0.05). The mean values of the tongue posture for the skeletal Class II group were significantly greater than those for the skeletal Class I group (*P* < 0.05). The mean value of the tongue length for the two groups did not exhibit any statistically significant differences (*P* > 0.05). In addition, the sagittal sectional area of the tongue (TsurA) in the skeletal Class I group was significantly larger than that in the skeletal Class II group (*P* < 0.05). It was suggested that patients in skeletal Class II group had lower tongue posture, and the tongue body was relatively small.

**Table 1:** Comparison of tongue measurements between the two groups

Group	Tongue posture (mm)							Tongue morphology	
	T1	T2	T3	T4	T5	T6	T7	TL (mm)	TsurA (mm^2^)
Skeletal class I (n = 45)	0.00 ± 0.00	0.09 ± 0.33	0.43 ± 1.32	0.68 ± 1.57	0.89 ± 1.86	0.49 ± 1.23	0.36 ± 0.64	62.37 ± 3.80	1236.75 ± 146.22
Skeletal class II (n = 45)	1.13 ± 1.55	2.35 ± 2.57	4.27 ± 3.38	6.05 ± 3.51	6.55 ± 4.23	6.14 ± 4.26	4.07 ± 3.38	60.69 ± 3.74	1019.62 ± 183.32
*t*	− 3.632	− 4.478	− 5.833	− 7.114	− 7.187	− 7.639	− 6.266	1.126	5.567
*P*	0.001	< 0.001	< 0.001	< 0.001	< 0.001	< 0.001	< 0.001	0.274	< 0.001

### Hyoid bone measurements in two groups

The hyoid bone measurement parameters of the two groups are summarized in Tables 2 and 3. The results of hyoid bone position measurement showed that H–H' and H–Y in the skeletal Class II group were larger than those in the Class I group (*P* < 0.05). However, other measurement parameters of hyoid bone position did not differ significantly between the two groups (*P* > 0.05). Comparing the mean hyoid bone morphology parameters between the two groups, the results showed that they did not differ significantly (*P* > 0.05). It was suggested that the position of the hyoid bone in skeletal Class II group was lower and located at the posterior-inferior place.

**Table 2:** Comparison of hyoid bone position between the two groups

Group	C3-H (mm)	H-Me (mm)	C3-Me (mm)	H–H' (mm)	H–X (mm)	H-Y (mm)	H-N-S (°)	H–S-Ba (°)
Skeletal Class I (n = 45)	31.88 ± 3.85	38.87 ± 8.07	68.85 ± 5.45	0.18 ± 4.30	24.82 ± 13.76	95.64 ± 6.65	57.68 ± 3.83	38.90 ± 5.51
Skeletal Class II (n = 45)	32.35 ± 3.78	37.19 ± 4.58	66.65 ± 6.56	2.85 ± 5.33	26.15 ± 8.48	100.08 ± 6.83	58.09 ± 4.55	37.00 ± 5.18
*t*	0.536	1.039	1.360	− 2.098	− 0.179	− 2.003	− 0.368	1.352
*P*	0.558	0.268	0.163	0.039	0.856	0.035	0.726	0.181

**Table 3:** Comparison of hyoid bone morphology between the two groups

Group	Ha W (mm)	Hp W (mm)	Hb L (mm)	HR (mm)	HL (mm)
Skeletal class I (n = 45)	18.33 ± 2.53	41.22 ± 4.55	37.62 ± 45.75	27.05 ± 2.41	34.32 ± 41.79
Skeletal class II (n = 45)	17.96 ± 2.73	40.41 ± 5.43	29.73 ± 4.12	26.59 ± 3.32	26.63 ± 3.39
*t*	0.865	0.626	1.016	0.988	1.085
*P*	0.384	0.515	0.313	0.321	0.282

### Tongue and hyoid bone measurements with different vertical facial patterns in each group

The descriptive statistics for the tongue measurements with different vertical facial patterns in the skeletal Class I group are shown in Table 4. There was no significant difference in the results of tongue position in the skeletal Class I group with different vertical facial patterns (*P* > 0.05). The tongue length in the skeletal Class I group with low angle was longer than that with average angle and high angle (*P* < 0.05), but the sagittal sectional area (TsurA) of the tongue with different vertical facial patterns did not exhibit any statistically significant differences (*P* > 0.05).

**Table 4:** Comparison of tongue measurements in skeletal Class I group with different vertical facial patterns

Type	Tongue posture (mm)							Tongue morphology	
	T1	T2	T3	T4	T5	T6	T7	TL (mm)	TsurA (mm^2^)
Low angle (n = 15)	0.00 ± 0.00	0.01 ± 0.03	0.98 ± 2.18	1.10 ± 2.43	1.09 ± 1.86	0.13 ± 0.05	0.08 ± 0.13	65.90 ± 1.78	1248.21 ± 182.16
Average angle (n = 15)	0.00 ± 0.00	0.15 ± 0.43	0.37 ± 1.45	0.91 ± 1.90	0.96 ± 2.15	0.50 ± 1.59	0.33 ± 0.97	62.37 ± 3.39	1243.33 ± 156.45
High angle (n = 15)	0.00 ± 0.00	0.01 ± 0.03	0.45 ± 0.83	0.36 ± 1.12	0.24 ± 0.69	0.59 ± 1.52	0.25 ± 0.83	59.63 ± 4.29	1220.01 ± 113.09
*F*	0.615	0.324	0.349	0.314	0.538	0.295	0.252	3.889	0.082
*P*	0.547	0.733	0.718	0.725	0.574	0.747	0.789	0.029	0.933

The results of hyoid bone measurement with different vertical facial types in the skeletal Class I group are given in Tables 5 and 6. The H-Me of the low-angle group was significantly larger than that of the average-angle group and the high-angle group (*P* < 0.05). However, other measurement parameters of hyoid bone position did not differ significantly among the three subgroups of facial growth patterns (*P* > 0.05). In addition, there was no significant difference among the three subgroups of facial growth patterns with respect to hyoid bone morphology (*P* > 0.05).

**Table 5:** Comparison of hyoid bone position in the skeletal Class I group with different vertical facial patterns

Type	C3–H (mm)	H–Me (mm)	C3–Me (mm)	H–H' (mm)	H–X (mm)	H–Y (mm)	H–N–S (°)	H–S–Ba (°)
Low angle (n = 15)	36.26 ± 6.25	56.03 ± 17.58	75.49 ± 4.68	1.32 ± 2.49	23.87 ± 3.93	99.80 ± 8.06	57.86 ± 1.99	37.47 ± 4.88
Average angle (n = 15)	31.96 ± 3.65	37.49 ± 4.06	69.61 ± 4.83	-0.48 ± 5.69	25.11 ± 6.17	95.93 ± 6.26	58.17 ± 3.32	39.96 ± 4.80
High angle (n = 15)	30.65 ± 3.13	37.22 ± 6.09	68.49 ± 6.78	1.30 ± 3.05	28.68 ± 28.39	97.78 ± 8.03	56.55 ± 5.67	36.69 ± 7.42
*F*	1.688	11.811	1.836	0.495	0.210	0.560	0.537	1.220
*P*	0.215	< 0.001	0.177	0.614	0.811	0.577	0.590	0.309

**Table 6:** Comparison of hyoid bone morphology in skeletal Class I group with different vertical facial patterns

Type	Ha W (mm)	Hp W (mm)	Hb L (mm)	HR (mm)	HL (mm)
Low angle (n = 15)	20.29 ± 2.44	43.31 ± 5.76	30.59 ± 4.30	28.46 ± 2.12	29.01 ± 0.65
Average angle (n = 15)	18.72 ± 2.59	41.36 ± 5.06	42.39 ± 56.22	27.69 ± 2.00	38.30 ± 51.44
High angle (n = 15)	17.87 ± 2.77	40.25 ± 3.85	27.80 ± 2.26	25.78 ± 3.07	25.92 ± 2.95
*F*	0.987	0.472	0.354	2.620	0.298
*P*	0.384	0.628	0.705	0.088	0.744

The results of tongue measurement with different vertical facial types in the skeletal Class II group are shown in Table 7. The descriptive statistics for the hyoid bone measurements with different vertical facial patterns in skeletal Class II group are given in Tables 8 and 9. The results showed that there was no significant difference in the position and morphology of tongue and hyoid bone in the skeletal Class II group with different vertical facial patterns (*P* > 0.05).

**Table 7:** Comparison of tongue measurements in skeletal Class II group with different vertical facial patterns

Type	Tongue posture (mm)							Tongue morphology	
	T1	T2	T3	T4	T5	T6	T7	TL (mm)	TsurA (mm^2^)
Low angle (n = 15)	1.06 ± 1.67	2.05 ± 2.58	3.56 ± 3.23	4.88 ± 3.86	5.14 ± 4.27	4.67 ± 3.84	4.00 ± 3.63	59.05 ± 5.90	914.98 ± 140.68
Average angle (n = 15)	1.29 ± 1.97	2.19 ± 2.48	3.83 ± 2.80	6.10 ± 3.33	6.55 ± 4.18	6.58 ± 4.61	4.23 ± 3.83	61.56 ± 4.21	1014.69 ± 191.90
High angle (n = 15)	0.00 ± 0.00	0.00 ± 0.00	0.42 ± 0.85	0.38 ± 1.14	0.24 ± 0.73	0.59 ± 1.52	0.27 ± 0.82	60.65 ± 4.06	1085.11 ± 178.22
*F*	0.211	0.0379	1.031	0.510	0.688	0.590	0.104	0.734	1.663
*P*	0.811	0.963	0.368	0.605	0.510	0.560	0.902	0.488	0.205

**Table 8:** Comparison of hyoid bone position in the skeletal Class II group with different vertical facial patterns

Type	C3-H (mm)	H-Me (mm)	C3-Me (mm)	H–H' (mm)	H–X (mm)	H-Y (mm)	H-N-S (°)	H–S-Ba (°)
Low angle (n = 15)	32.81 ± 4.58	38.78 ± 3.54	66.10 ± 11.09	4.86 ± 4.77	30.65 ± 8.66	100.33 ± 7.40	59.07 ± 4.78	38.78 ± 6.54
Average angle (n = 15)	32.41 ± 3.12	36.83 ± 6.55	68.18 ± 6.84	2.56 ± 5.79	27.02 ± 9.00	99.43 ± 6.68	57.93 ± 5.55	36.48 ± 7.03
High angle (n = 15)	32.12 ± 4.82	37.26 ± 3.28	67.46 ± 7.08	1.81 ± 5.26	22.86 ± 7.66	101.80 ± 8.61	57.88 ± 2.88	36.90 ± 4.41
*F*	0.058	0.297	0.170	0.598	1.632	0.338	0.144	0.302
*P*	0.943	0.745	0.845	0.556	0.211	0.716	0.867	0.742

**Table 9:** Comparison of hyoid bone morphology in skeletal Class II group with different vertical facial patterns

Type	Ha W (mm)	Hp W (mm)	Hb L (mm)	HR (mm)	HL (mm)
Low angle (n = 15)	17.43 ± 2.18	41.09 ± 2.77	30.98 ± 3.65	27.98 ± 4.00	28.29 ± 3.97
Average angle (n = 15)	18.29 ± 3.03	40.78 ± 6.34	29.35 ± 3.76	26.07 ± 2.57	26.12 ± 2.80
High angle (n = 15)	18.05 ± 2.92	39.40 ± 5.89	29.71 ± 5.21	26.68 ± 4.25	26.62 ± 4.07
*F*	0.207	0.232	0.342	0.743	0.925
*P*	0.814	0.794	0.713	0.484	0.407

## Discussion

The tongue is an important muscular structure in the oral cavity that determines the dental arch form. The forces exerted by tongue play a vital role in the guidance of dental arch formation and the establishment of the jaw relationship [9]. Therefore, the altered position of the tongue may cause imbalance in the forces, which may result in alterations in the surrounding dentoalveolar structures and craniofacial growth. The hyoid bone is a unique bone that is not articulated with other bones and that floats in connection with ligaments and muscles. The position of the hyoid bone is determined by the conjoint action of the muscles and ligaments that are attached to structures such as pharynx, mandible, and cranium. The hyoid bone moves during respiration, mastication, deglutition and phonation, and it is closely related to tongue due to its surrounding musculature [10]. Therefore, it is suggested that the tongue and hyoid bone may be related to the mandibular position and morphology.

For patients with skeletal class II malocclusion, craniofacial dysplasia is often accompanied by abnormal tongue and hyoid bone. The present study established a tongue posture study model on CBCT images, and comprehensively studied the positional and morphological characteristics of tongue in patients with skeletal class II malocclusion. The results found that the patients in the skeletal Class II group had lower tongue posture than those in the skeletal Class I group and that the tongue body was relatively small. This finding is in agreement with a study by Ashish Chauhan et al. [5]. It may be related to the mandible retrusion in patients with skeletal class II malocclusion, which leads to lower tongue posture. In addition, a lower tongue posture may result in a part of the root of the tongue falling into the nasopharynx tube, making the volume of the tongue smaller. The position of hyoid bone reflects the tensions of muscles, ligaments and fascia attached to it, which also changes the position and function of hyoid bone [11]. The geniohyoid and mylohyoid muscles are attached near the symphysis of the mandible and affect the position of the hyoid bone by tongue movements and mandibular movements [10]. Additionally, alternations of hyoid bone position after orthognathic operations have been reported. The hyoid position moves upward after mandibular advancement surgery and downward after mandibular setback surgery [12, 13]. Mortazavi et al. revealed that in skeletal class III with mandibular prognathism, the hyoid bone is positioned more anteriorly than in class I and II. In skeletal class II with mandibular deficiency, this bone is positioned more superiorly and posteriorly than the two other groups [14]. In the present study, the results show that the vertical H–Y and H–H of skeletal class II malocclusion patients were significantly greater than that of skeletal class I malocclusion patients, while there was no significant difference in sagittal hyoid position measurement indexes. This finding may be due to the downward and posterior growth of mandible of patients with skeletal class II malocclusion (i.e., clockwise growth type), which leads to the change of hyoid position.

It has been stated that the stability of the position of hyoid bone was affected by the inclination of the mandible, craniofacial postural changes and was interrelated with facial type [15]. However, Kocakara et al. [16] stated that the position of the hyoid bone was not greatly affected by vertical facial development. In this study, TL and H-Me in skeletal Class I group with low angle were significantly larger than those with average angle and high angle. This finding can be explained by the fact that the mandible in the low-angle group has a trend of horizontal forward growth, which leads to the increase in the relative length of the mandible in the sagittal direction and the length of the tongue body. However, there was no significant difference in the position and morphology of tongue and hyoid bone in skeletal Class II group with different vertical facial patterns.

## Conclusion

1. Patients with skeletal class II malocclusion have lower tongue posture, smaller tongue body, and posterior inferior hyoid bone position than skeletal Class I patients.
2. The position and morphology of tongue and hyoid bone were not greatly affected by vertical facial development in skeletal class II patients.
3. Skeletal class I patients with lower angle vertical type have longer TL and larger H-Me than the patients with average angle and high angle, which indicated that the length of mandibular body in patients with horizontal growth type is longer.

## Data Availability

The datasets used and/or analysed during the current study are available from the corresponding author on reasonable request.

## Abbreviations

CBCT: Cone beam computed tomography
FH plane: The Frankfort horizontal plane
DICOM: Digital imaging and communications in medicine
N: Nasion
ANS: Anterior nasal spine
PNS: Posterior nasal spine
Ba: Basion
S: Sella
N: Nasion
Ba: Basion
TT: Tip of tongue
EP: The deepest point of the epiglottis
C3: The most antero-inferior point on the corpus of the third cervical vertebra
Me: Menton
H: The most antero-superior point on the body of the hyoid bone
H’: The intersection point of vertical line of C3-Me through H with line C3-Me
